# The comparative study for survival outcome of locally advanced cervical cancer treated by neoadjuvant arterial interventional chemotherapy or intravenous chemotherapy followed by surgery or concurrent chemoradiation

**DOI:** 10.1186/s12957-022-02859-w

**Published:** 2022-12-07

**Authors:** Yi Sun, Gailing Li, Panpan Hai, Yuan Cao, Pin Han, Yuchen Liu, Jing Wen, Yuanpei Wang, Xiaoran Cheng, Fang Ren

**Affiliations:** Department of Gynecology, The First Affiliated Hospital of Zhengzhou University, Zhengzhou, 450000 China

**Keywords:** Locally advanced cervical cancer, Neoadjuvant arterial chemotherapy, Neoadjuvant intravenous chemotherapy, Concurrent chemoradiotherapy, Surgery

## Abstract

**Objective:**

This study aimed to compare the survival outcome of 3 different treatment groups (arterial interventional chemotherapy or intravenous chemotherapy or concurrent chemoradiotherapy) for locally advanced cervical cancer.

**Methods:**

A total of 187 patients with pathological stage IB3–IIB cervical cancer (cervical squamous cell carcinoma, adenosquamous carcinoma, or adenocarcinoma) hospitalized in the First Affiliated Hospital of Zhengzhou University from January 2013 to May 2019 were included. Therefore, this article is a retrospective study. We collected data from all eligible patients. And all according to the treatment methods at that time, they were divided into three subgroups: (1) 40 patients who received neoadjuvant arterial interventional chemotherapy + surgery + postoperative chemotherapy (IA-NAC + RS), (2) 63 patients who received neoadjuvant intravenous chemotherapy + surgery + postoperative chemotherapy (IV-NAC + RS), (3) 84 patients who only received concurrent chemoradiotherapy (CCRT). Notably, 108 of these patients met the 5-year follow-up period, and 187 patients met the 3-year follow-up period only. Consequently, we compared 5-year survival and 3-year survival separately. The prognosis (5-year survival and 3-year survival) of the three groups and the chemotherapy efficacy, intraoperative blood loss, operation time, and postoperative pathological risk factors of different subgroups were compared.

**Results:**

(1) There were no significant differences in the 3-year overall survival (OS) rate, 3-year progression-free survival (PFS) rate, 5-year OS rate, and 5-year PFS rate among the three subgroups (*p* > 0.05). (2) The chemotherapy response rates of IA-NAC+RS group (37.5%) and IV-NAC+RS group (25.4%) were comparable (*p* > 0.05). (3) The intraoperative blood loss in the IA-NAC+RS group (average 92.13±84.09 mL) was significantly lower than that in the IV-NAC+RS group (average 127.2±82.36 mL) (*p* < 0.05). (4) The operation time of the IA-NAC+RS group (average 231.43±63.10 min) and the IV-NAC+RS group (average 219.82±49.11 min) were comparable (*p* > 0.05). (5) There were no significant differences between the IA-NAC+RS group and IV-NAC+RS group in pathological lymph node metastasis, parametrial invasion, and involvement of lymphovascular space (*p* > 0.05).

**Conclusions:**

Neoadjuvant chemotherapy combined with surgery had the same long-term survival benefit as concurrent chemoradiotherapy.

## Introduction

The incidence rates of cervical cancer are only to that of breast cancer worldwide, and the leading cause of cancer deaths among women in developing countries [1]. In China, it is estimated that there are approximately about 98,900 new cases of cervical cancer and 30,500 deaths of cervical cancer each year, ranking first among gynecological malignant tumors in China [2, 3]. With the popularization of cervical cancer screening and the improvement of treatment technology, the long-term prognosis of cervical cancer has been greatly improved [4–6].

The concurrent chemoradiotherapy (CCRT) is recommended as the primary treatment for locally advanced cervical cancer (LACC) in the 2022 NCCN guidelines [7]. However, CCRT can lead to an array of radiotherapy-related complications, such as radiation proctitis and radiation cystitis. At the same time, the destruction of ovarian function caused by radiotherapy can also have a great negative impact on the quality of life of patients [8–10]. Neoadjuvant chemotherapy (NACT) combined with radical surgery (radical hysterectomy + pelvic lymph node dissection) has become an effective alternative for the treatment of patients with LACC [11, 12]. The feasibility and effectiveness of this treatment in LACC have been validated in several clinical trials [13, 14]. Neoadjuvant chemotherapy mainly includes arterial interventional chemotherapy and intravenous chemotherapy. Relevant studies have confirmed that arterial interventional chemotherapy can reduce postoperative pathological risk factors, and the 5-year overall survival (OS) rate of patients receiving arterial interventional chemotherapy is better than those receiving intravenous chemotherapy [15–17], but the current evidence is limited.

Preoperative arterial chemotherapy for locally advanced cervical cancer can be traced back as far as the 1960s. The first report on IA-NAC was published in 1950 by Bierman et al. [18], and its use in the gynecological field was reported in 1952 [19]. IA-NAC is employed to obtain higher tissue drug concentrations by direct infusion of anticancer agents into the feeding artery of a tumor to maximize the anticancer activity and minimize adverse effects related to drugs entering the systemic circulation [20]. Possible complications in arterial chemotherapy in some patients are mainly post-embolization syndrome, such as fever, pain, nausea, and vomiting, which can improve faster after symptomatic support treatment with anti-inflammatory, analgesic, and rehydration [21].

This study mainly aimed to compare the long-term outcomes of different subgroups receiving neoadjuvant arterial interventional chemotherapy + surgery + chemotherapy (IA-NAC+RS), neoadjuvant intravenous chemotherapy + surgery + chemotherapy (IV-NAC+RS), or CCRT in a retrospective study of LACC. These results may provide guidance for clinicians to choose therapeutic methods of LACC.

## Material and methods

### Patient selection and ethical approvement

At the beginning of the study, we included a total of about 300 patients who were diagnosed as LACC in the First Affiliated Hospital of Zhengzhou University from January 2013 to May 2019. However, some patients were subsequently excluded from the group because they did not meet the inclusion and exclusion criteria or there was a serious lack of information. And finally, we included 187 patients who met the requirements. Patients in the NACT subgroup did not receive radiation therapy after the follow-up study. Some patients did not need radiotherapy based on postoperative pathological evaluation, and some patients refused radiotherapy for personal reasons.

The inclusion criteria are as follows: (1) clinical stage (FIGO 2018) IB3-IIB and lesion diameter ≥4cm and (2) squamous cell carcinoma, adenosquamous carcinoma, and adenocarcinoma. The exclusion criteria are as follows: (1) the presence of contraindications to radiotherapy and chemotherapy such as abnormal liver, kidney, and bone marrow function; (2) history of tumors; (3) the NACT (IA-NAC+RS and IV-NAC+RS) group received radiotherapy; (4) pregnancy, heart disease, and other comorbidities.

This study was a retrospective study. We selected patients between January 2013 and May 2019. Among this group of patients, we selected the appropriate patients for enrollment based on the inclusion criteria. Then collect relevant information (especially the treatment modalities at the time), and the allocation of patients was based on the discretion of the treating physicians at that time. The patients were divided into 3 subgroups according to the treatments: 40 patients who received IA-NAC+RS, 63 patients who received IV-NAC+RS, and 84 patients who received CCRT. The demographic characteristics (e.g., age, tumor diameter, tumor stage, pathological type, and degree of differentiation) were similar in these three subgroups. This study was approved by the Ethics Committee of the First Affiliated Hospital of Zhengzhou University.

### Treatments

#### IA-NAC+RS

All patients received 1–2 times arterial interventional chemotherapy (paclitaxel combined with platinum), then underwent laparoscopic radical hysterectomy + pelvic lymph node dissection, and received 2–5 times postoperative chemotherapy (paclitaxel combined with platinum). The times of NACT depended on the patient’s tolerance and clinical response. The specific operation of arterial interventional chemotherapy was as follows. Under the surveillance of digital subtraction angiography, the right femoral artery was punctured using the Seldinger technique. Cobra catheters were introduced for cannulation and angiography of bilateral internal iliac arteries and uterine arteries, respectively. Diluted platinum (80 mg/m^2^) was injected into the uterine artery, and gelatin sponge particles were used to embolize the anterior trunk of the internal iliac artery. Repeated angiography was successful when the blood flow of the target artery disappeared. After the operation, the catheter was extubated. After treatment, the catheters were removed, and sandbags were placed to apply firm pressure to the groin for 6 h. Hydration with normal saline and 5% dextrose was started from 3h before IA-NAC and was continued to maintain a urine output >100 ml/h for 24 h.

#### CCRT

Radiotherapy consisted of external beam irradiation and intracavitary brachytherapy with concurrent chemotherapy (PT, 4–6 cycles). The interval between each chemotherapy treatment is 3–4 weeks. The entire pelvis was externally irradiated (1.8 or 2 Gy) 23 to 25 times, totaling 45 to 50 Gy, according to Radiation Therapy Oncology Group guidelines. Brachytherapy was given at point A with the high dose rate (HDR) of 6 Gy per session for a total of 5 sessions (once a week).

### IV-NA+RS

All patients received 1–2 times neoadjuvant intravenous chemotherapy (paclitaxel combined with platinum), then followed by laparoscopic radical hysterectomy + pelvic lymph node dissection, and 2–5 times of paclitaxel combined with platinum chemotherapy after operation. The times of neoadjuvant chemotherapy depended on the patient’s tolerance and clinical response. During the treatment, patients were treated with paclitaxel combined with platinum. Of these, paclitaxel was 135–175 mg/m^2^. Platinum included cisplatin (30–40 mg/m^2^) and loplatin (30–40 mg/m^2^).

#### About neoadjuvant chemotherapy

Patients on neoadjuvant chemotherapy were evaluated for status before the next chemotherapy, and some patients responded well enough to undergo surgery directly after 1 chemotherapy session. For patients with lesion reduction after neoadjuvant chemotherapy, chemotherapy could be continued another neoadjuvant chemotherapy. In our study, 29 people in the IA-NAC+RS group had only 1 neoadjuvant chemotherapy and 46 people in the IV-NAC+RS group had only 1 neoadjuvant chemotherapy.

#### Treatment after surgery

This fraction of patients treated with neoadjuvant chemotherapy was treated with 3–5 more sessions of chemotherapy (paclitaxel combined with platinum) after surgery. The interval between each chemotherapy treatment is 3–4 weeks. In principle, radiotherapy should be added to patients with pelvic lymph node metastasis, cervical myometrial invasion greater than 1/2, tumor thrombus in vessels, and parametrial cancer invasion. In this article, patients with high-risk positive postoperative pathology have communicated with the patients and their families, who refused radiotherapy for personal reasons.

### Judgment of curative effect

In this study, the method of direct observation and measurement of tumor lesion size was adopted, and some patients were evaluated concerning vaginal ultrasound or CT or MRI. Tumor response to NACT and chemoradiation was assessed according to the World Health Organization criteria [22]. Complete response (CR) was defined as the complete disappearance of the tumor. Partial response (PR) was defined as a decrease in tumor size of 50% or more. Stable disease (SD) was defined as a reduction in tumor size of less than 50% or tumor enlargement less than 25%. Progressive disease (PD) was defined as an increase more than 25% in tumor volume or the appearance of new lesions. Both CR and PR were defined as treatment-sensitive responses. Both SD and PD were defined as treatment-invalid.

### Follow-up

After completion of the entire treatment, all patients were evaluated every 3 months for the first 2 years and every 6 months from 2 to 5 years. Inspections during follow-up included pelvic MRI, urinary ultrasound and liver and kidney ultrasound, cervical smear, X-ray, and tumor markers. The primary endpoints of the study included 5-year progression-free survival (PFS) and 5-year OS, and secondary endpoints included 3-year PFS and 3-year OS. PFS was the time from the date of treatment to tumor recurrence or the last follow-up time; OS was the time from the day of treatment to death or last follow-up.

### Statistical analysis

We used SPSS 26.0 for the statistical analysis. Quantitative data were expressed as mean ± standard deviation after the normality test which obeyed normal distribution, and non-normal distribution was expressed as a median. Qualitative data were compared using the *χ*^2^ test, continuous corrected chis-square test, or Fisher’s test. Survival was calculated using the Kaplan-Meier method and compared using the log-rank test. And *p*<0.05 was considered statistically significant.

## Results

### Baseline characteristics of patients

A total of 187 eligible patients were included in the study. The baseline characteristics of the included patients are shown in Table 1. There were no significant differences in age, clinical stage, tumor size, differentiation degree, and other characteristics between different subgroups (*p* > 0.05).

**Table 1:** Baseline characteristics for all patients

	IACT + RS	IVCT + RS	CCRT	*p*	*χ*^2a^
Age (years)	48.33±10.44	52.41±10.36	52.90±10.79	0.094	-
FIGO stage, *n* (%)					
IB2	13 (32.5%)	17 (27.0%)	20 (23.8%)	0.888	1.138
IIA2	19 (47.5%)	31 (49.2%)	43 (51.2%)		
IIB	8 (20.0%)	15 (23.8%)	21 (25.0%)		
Pathological type, *n* (%)					
Squamous cell carcinoma	36 (90.0%)	59 (93.7%)	79 (94.0%)		
Non-squamous cell carcinoma	4 (10.0%)	4 (6.3%)	5 (6.0%)	0.889	0.703
Differentiation, *n* (%)					
Well-differentiated and moderately differentiated	31 (77.5%)	52 (82.5%)	67 (79.8%)	0.411	0.814
Poorly differentiated	9 (22.5%)	11 (17.5%)	17 (20.2%)		

^a^The *χ*^2^ is a statistic in nonparametric tests and is mainly used in nonparametric statistical analysis. It is used to test the correlation of the data. If the significance of the *χ*^2^ is less than 0.05, it means that the two variables are significantly correlated

### Postoperative pathological factors in different neoadjuvant chemotherapy subgroups

The *χ*^2^ test was used to compare the pathological characteristics that may affect the prognosis, and it was found that there were no significant differences in lymph node metastasis, positive incision margin, and lymphovascular space involvement between the two subgroups (*p* > 0.05, Table 2).

**Table 2:** Intraoperative pathology in neoadjuvant chemotherapy subgroups

	Lymph node metastasis	LVSI	Positive margins
IA-NAC+ RS	7 (17.5%)	3 (7.5%)	1 (2.5%)
IV-NAC+ RS	8 (12.7%)	7 (11.1%)	2 (3.2%)
*p*	0.501	0.793	1

### Survival outcomes of patients

**Fig. 1:**
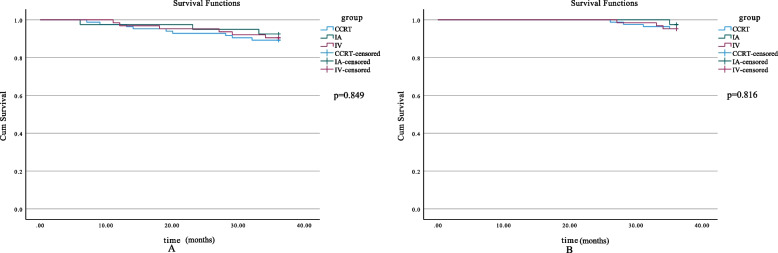
Three-year survival curves. **A** 3-year PFS of all patients in different subgroups (*p* = 0.849). **B** 3-year OS of all patients in different subgroups (*p* = 0.816). OS, overall survival; PFS, progression-free survival

**Fig. 2:**
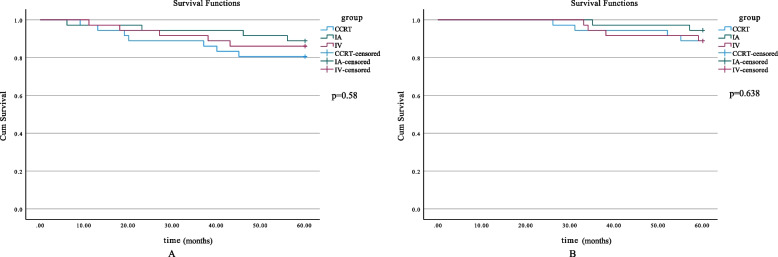
Five-year survival curves. **A** 5-year PFS of all patients in different groups (*p* = 0.58). **B** 5-year OS of all patients in different subgroups (*p* = 0.638). OS, overall survival; PFS, progression-free survival

The Kaplan-Meier method was used to calculate the OS and PFS. There were 40, 63, and 84 patients reaching 3-year follow-up in three subgroups, respectively. There were 36 patients in the 3 groups who met the 5-year follow-up period, respectively. The results showed that the 3-year PFS rates did not differ among three subgroups (*p* = 0.849), which were 92.5%, 90.5%, and 89.3% respectively. There were also no significant differences in the three subgroups regarding 3-year OS rates (*p* = 0.816), which were 97.5%, 95.2%, and 95.2% respectively (Fig. 1). Similar findings were found in three subgroups regarding 5-year OS rates (94.4%, 88.9%, 88.9% respectively) and 5-year PFS rates (88.9%, 86.1%, 80.6% respectively) (*p* > 0.05) (Fig. 2).

**Fig. 3:**
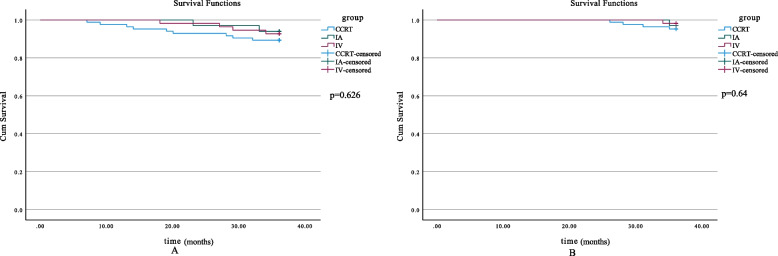
Three-year survival curves (eliminate patients with lymph node metastases). **A** 3-year PFS of all patients in different subgroups (*p* = 0.626). **B** 3-year OS of all patients in different subgroups (*p* = 0.64). OS, overall survival; PFS, progression-free survival

**Fig. 4:**
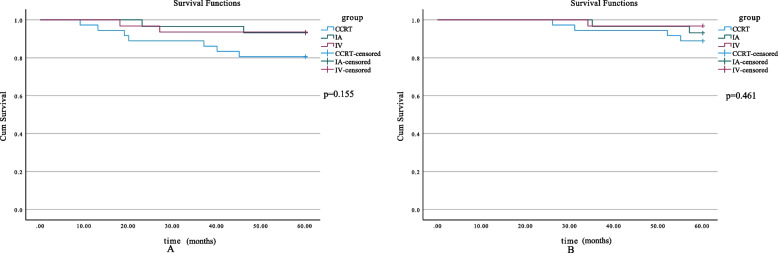
Five-year survival curves (eliminate patients with lymph node metastases). **A** 5-year PFS of all patients in different subgroups (*p* = 0.155). **B** 3-year OS of all patients in different subgroups (*p* = 0.461). OS, overall survival; PFS, progression free survival

It is worth noting that, as shown in Table 2, 7 patients in the IA-NAC+ RS subgroup who met the 3-year and 5-year follow-up periods had lymph node metastasis. Eight patients in the IV-NAC + RS group who met the 3-year follow-up period had lymph node metastasis, and a total of 5 patients who met the 5-year follow-up period had lymph node metastasis. However, according to FIGO 2018, the cases who had lymph node-positive were classified as stage IIIC due to bad prognosis. The aim of this study was to investigate the effect of adjuvant chemotherapy among LACC (IB3 and IIB). Remaining the stage IIIC in the study was not reasonable. And in our study, none of the patients included in the CCRT group had lymph node metastases as verified by imaging. Therefore, after excluding these patients mentioned above, we reperformed the survival analysis of the three subgroups. The differences of the 3-year PFS rates in three subgroups remained unsignificant (*p* = 0.626), which were 93.9%, 92.7%, and 89.3%. The 3-year OS rates of these three subgroups were 97.0%, 98.2%, and 95.2%, and the differences were also not statistically significant (*p* = 0.64) (Fig. 3). There were no significant differences regarding 5-year PFS rates (93.1%, 95.5%, 80.6%) and 5-year OS rates (93.1%, 96.8%, 88.9%) among the three subgroups (*p* > 0.05) (Fig. 4).

Among the patients with postoperative pathology suggestive of lymph node metastasis, two patients in the IA-NAC+ RS group relapsed (one within the 3-year follow-up period and one within the 5-year follow-up period), both with abnormally high swelling markers. Two patients in the IV-NAC+ RS group died during the 3-year follow-up period and two patients died during the 5-year follow-up period. Among the patients in the CCRT group, 12 patients relapsed (9 patients relapsed during the 3-year follow-up period) and 6 patients died of relapse.

### Surgical conditions of different neoadjuvant chemotherapy groups

All patients underwent radical hysterectomy + pelvic lymphadenectomy (+ oophorectomy or ovarian transposition). The operative time in the IA-NAC+RS group was 120–460 min, and the mean time was 231.43±63.10 min. Intraoperative bleeding of this group was 20–400 mL, with an average of 92.13±84.09 mL. The operation time of the IV-NAC+RS group was 130–311min, and the average time was 219.82±49.11min. Intraoperative bleeding was 20–300 mL, with an average of 127.2±82.36 ml. There was no significant difference in operation time between these two subgroups (*p* > 0.05), but there was a significant difference in the amount of blood loss between the two subgroups (*p*<0.05).

### Chemotherapy efficacy of different neoadjuvant chemotherapy groups

In the two subgroups receiving NACT, no patients developed PD. The clinical response rate of IA-NAC+ RS group (37.5%) was comparable to that of IV-NAC+ RS group (25.4%), and the difference was not statistically significant (*p* = 0.192, Table 3).

**Table 3:** The efficacy in neoadjuvant chemotherapy subgroups

	*n*	Clinically effective	Clinically ineffective	*p*	*χ*^2^
IA-NAC+ RS	40	15 (37.5%)	25 (62.5%)	0.192	1.704
IV-NAC+ RS	63	16 (25.4%)	47 (74.6%)		

The imaging-based rate of no residual disease was 27.5% in the IA-NAC+RS group, which was significantly higher than that in the IV-NAC+RS group (11.1%), and the difference was statistically significant (*p*=0.033, Table 4).

**Table 4:** Lesion reduction in neoadjuvant chemotherapy subgroups

	*n*	No residual disease	Residual disease	*p*	*χ*^2^
IA-NAC+ RS	40	11 (27.5%)	29 (72.5%)	0.033	4.557
IV-NAC+ RS	63	7 (11.1%)	56 (88.9%)		

## Discussion

Patients with LACC are usually accompanied by high-risk prognostic factors such as positive pelvic nodes and large tumor volume [23]. For patients diagnosed as stage IB3-IIB and with tumor diameter greater than 4 cm, the long-term effects of direct surgery or radiotherapy or postoperative radiotherapy are not ideal [24]. Currently, CCRT is recommended by NCCN as the first choice for LACC. CCRT significantly improves survival outcomes of LACC. Unfortunately, CCRT-induced treatment complications still remain the challenge for patient’s quality of life [25, 26]. In recent years, NACT+RS has been used as an effective method for the treatment of LACC [12, 27].

The survival benefit of neoadjuvant chemotherapy combined with surgery in patients with LACC has been proven by some researchers. Marchetti et al. [28] proved that patients obtained similar benefit from both modalities (NACT + RS or CCRT) regarding OS in a meta-analysis. However, Yin et al. [29] revealed that NACT+RS (88.67%) could significantly improve the 5-year OS rate compared to the CCRT (64.37%). Furthermore, the 5-year disease-free survival rates were also significantly improved by the NACT+RS (85.00% versus 52.94%) in that study. In our study, the OS and PFS of the three subgroups were not statistically different. The NACT group had the same survival outcomes as the CCRT group.

In addition, in our study, postoperative pathological reports revealed that there were 7 and 8 patients diagnosed with lymph node metastasis in the IA-NAC+RS group and the IV-NAC+RS group, respectively. These patients were upgraded to stage IIIC according to postoperative pathological staging. And in our study, none of the patients included in the NACT group had lymph node metastases as verified by imaging. After excluding these patients, there was no statistical difference in OS and PFS among the three subgroups. This again confirms that both NACT and CCRT have the same survival benefits.

The approaches of neoadjuvant chemotherapy include arterial interventional chemotherapy and intravenous chemotherapy. In this study, arterial interventional chemotherapy was superior to intravenous chemotherapy in terms of surgery and long-term benefits. As for surgery, the intraoperative blood loss in the IA-NAC+RS group was less than that in the IV-NAC+RS group. This is mainly because preoperative arterial chemotherapy can better achieve the purpose of tumor shrinkage and blood supply reduction [30]. The operation time of the IA-NAC+RS group (average 231.43±63.10 min) and the IV-NAC+RS group (average 219.82±49.11 min)were comparable (*p* > 0.05). But the operation time in the IA-NAC+RS group was slightly higher than that one. Considering that the tissue was in a state of congestion during the operation after interventional chemotherapy, it was related to the fibrosis of the parametrial tissue caused by embolization.

In addition, we also compared the pathological features that may affect the prognosis and found that there was no significant difference between the two groups (IA-NAC+RS group versus IV-NAC+RS group) in lymph node metastasis, positive resection margin, and lympho-vascular space involvement (*p* > 0.05, Table 2), indicating that arterial chemotherapy did not increase the risk of prognosis compared with intravenous chemotherapy. In terms of long-term benefits, arterial interventional chemotherapy can directly apply chemotherapeutic drugs to the lesions. It can reduce the first-pass effect of the drug and increase the drug concentration of local tumors, thereby improving the efficacy of the drug [20]. It can not only quickly and accurately kill tumor cells for reducing tumor volume, but also reduce the retention of chemotherapy drugs in the body which can reduce the occurrence of gastrointestinal reactions in patients’ risk of adverse reactions such as liver and kidney insufficiency and bone marrow suppression [31]. Aok [32] and Yamada [33] achieved an effective rate of 80–91% in the treatment of LACC through arterial interventional chemotherapy. In our study, the effective rate in the IA-NAC+RS group (37.5%) was comparable to that in the IV-NAC+RS group (25.4%). Previous studies have shown that interventional chemotherapy can promote tumor cell apoptosis; inhibit tumor growth, invasion, and metastasis; and improve prognosis by regulating the expressions of VEGF, Caspase-9, TNFAIP8, and Prdx4 [34–37]. Also, the rate of no residual disease confirmed by imaging in the IA-NAC+RS group (27.5%) was significantly higher than that in IV-NAC+RS (11.1%). The above conclusions further verify that neoadjuvant chemotherapy is effective in the treatment of local cervical cancer, and the effect of arterial chemotherapy may be better than that of intravenous chemotherapy.

Our article has the following shortcomings. The number of patients included in the article was 187, which is a small number of patients. In addition, the side effects of different treatment regimens are required when performing long-term prognostic analysis of patients. But because this study was retrospective, some information was lost. Nevertheless, we summarized the side effects mentioned in the relevant studies. Studies have confirmed that CCRT can not only cause gastrointestinal and blood system toxicity but also cause more severe radiation enteritis and radiation cystitis [28, 34, 38]. Another point is that CCRT can lead to menopausal symptoms, sexual dysfunction, and irreversible damage to pelvic organs [4, 39]. It is essential to pay attention to the fact that radiation damage caused by radiotherapy is inevitable and that the pain caused by this radiation damage (radiation cystitis and radiation proctitis) cannot be improved. NACT group can effectively avoid radiation damage. Of course, there are some complications of chemotherapy, mainly hematologic toxicity and gastrointestinal [28]. However, both gastrointestinal and blood toxicity can be effectively prevented and treated with drugs. In addition, some very minor complications (fever and pain) were observed with arterial chemotherapy in our study. Thirteen patients in our study presented with abdominal pain, and six of them had severe pain, which improved after symptomatic management with analgesic drugs; one patient presented with fever (low afternoon fever) lasting for 1 week, which improved after symptomatic management. No other serious complications were seen in our study.

For patients with LACC, NACT combined with surgery (postoperative adjuvant chemotherapy) has the same survival benefit compared with CCRT. At the same time, based on the same survival benefit of interventional arterial chemotherapy and intravenous chemotherapy, interventional arterial chemotherapy can achieve better surgical. For limited included patients in our retrospective study, the above conclusions should be drawn cautiously. The choice of treatment for LACC needs to be further explored through a prospective randomized controlled study.

## Data Availability

The datasets analyzed during the current study are available from the corresponding author on reasonable request.
